# Co‐Designing a Prototype Education and Training Intervention for Healthcare Professionals Working in Community Laryngectomy Care: An Experience‐Based Co‐Design Study

**DOI:** 10.1111/hex.70870

**Published:** 2026-09-14

**Authors:** Laura‐Jayne Watson, Linda Sharp, David W. Hamilton, Vicky Thornton, Joanne M. Patterson

**Affiliations:** ^1^ Speech & Language Therapy/Institute of Population Health South Tyneside and Sunderland NHS Foundation Trust/University of Liverpool Sunderland/Liverpool UK; ^2^ Population Health Sciences Institute Newcastle University Centre for Cancer, Newcastle University Newcastle‐upon‐Tyne UK; ^3^ Head and Neck Surgery Freeman Hospital Newcastle upon Tyne UK; ^4^ Institute of Population Health University of Liverpool Liverpool UK; ^5^ School of Allied Health Professions & Nursing, Institute of Population Health/Liverpool Head and Neck Centre University of Liverpool Liverpool UK

**Keywords:** co‐design, community healthcare, education, laryngectomy, training

## Abstract

**Introduction:**

Evidence indicates that people with a laryngectomy often perceive community healthcare professionals as less knowledgeable than hospital‐based clinicians. Some individuals also report feeling more skilled than the professionals supporting them at home. This highlights a substantial gap in healthcare professional education and training, with significant safety implications for people with a laryngectomy, including reduced confidence in care, mistrust in healthcare professionals, and risks to continuity of care. To address this, the present research describes co‐design of a prototype education and training package for community healthcare professionals.

**Methods:**

Informed by the principles of Experience Based Co‐Design and underpinned by the Medical Research Council's guidance for Complex Intervention Development we ran prioritisation events followed by three iterative workshops with people with a laryngectomy, family members and healthcare professionals to develop the prototype education and training package. A digital expert supported all workshops. A combination of content and thematic analysis was used to analyse workshop data.

**Results:**

Twenty‐eight participants took part in the prioritisation events and workshops: eight people with a laryngectomy, five family members and fifteen healthcare professionals. All participants agreed the highest priority need for developing a digital solution to community‐based education and training. Preliminary programme theory, logic model and prototype education and training package were collaboratively designed. The education and training prototype includes ten agreed content topics (e.g. anatomy, communication), as well as design and implementation considerations.

**Conclusion:**

Through equal partnership working with key stakeholders, a prototype education and training package has been designed. The next stage is operationalisation, including co‐production of content/supporting resources and building of the digital platform, and refinement.

**Lived Experience Contribution:**

Lived experience was embedded throughout the study, shaping both its direction and delivery. Individuals with lived experience contributed as members of the research steering group, informing the overall study design and ensuring its relevance from the outset. A dedicated patient and public involvement group supported key stages of the project, including the development of the participant recruitment strategy, creation of resources for co‐design, and planning of the co‑design workshops. Their insights also informed the analysis of findings and the refinement of the final prototype, ensuring that the intervention remained grounded in the realities, priorities, and expertise of those directly affected. People with a laryngectomy and their families also took part in co‐designing the prototype.

## 1 Introduction

Laryngectomy is complete surgical removal of the larynx (voice‐box), typically undertaken as a treatment for advanced stage laryngeal cancer. After laryngectomy, people must acquire a complex set of new, lifelong skills including managing a permanent altered airway, establishing alternative communication, and adapting to changes in swallowing and physical appearance [1, 2]. The need to acquire complex new skills to support everyday functioning and ensure safety (both from an airway and communication perspective) can have a substantial impact on psychological wellbeing, adjustment and quality of life [3]. These challenges are further intensified by evolving models of postoperative care, such as enhanced recovery after surgery (ERAS) pathways, which prioritise earlier discharge and recovery in community settings [4, 5, 6]. This puts increased pressure on community healthcare professionals (HCPs) such as General Practitioners (GPs), District Nurses (DNs) and Pharmacists, who tend to be generalists rather than specialists, to support people with a laryngectomy (PwL).

Qualitative research suggests that many community HCPs, have limited experience and training in laryngectomy care [7]. From the perspective of PwL, hospital‑based clinicians are consistently perceived as having greater knowledge and expertise than community HCPs [8, 9, 10, 11, 12], and some individuals and families described feeling more knowledgeable and skilled than the HCPs supporting them at home [8, 9]. Collectively, these findings highlight a significant gap in education and training, with implications for safety, trust, and continuity of care following discharge into the community. This potential safety gap is exacerbated by recent research critically examining education and training, which highlighted a lack of consistency in education and training resource content relating specifically to this, delivery, evaluation, and implementation [13]. Consequently, there is a need to better understand how services can support PwL and their families to live, adjust, and adapt to life following laryngectomy within the community. Central to this is the development of targeted, sustainable education and training for community HCPs.

To ensure education and training resources are truly representative of PwL and their needs, it is essential that lived experience informs and underpins all stages of their development and delivery. The use of co‐design approaches in intervention development is well established as a means of making patient‐centred services a reality [14], while also shifting the focus away from interventions designed solely on professional judgement towards those informed by lived experience [15]. There is growing evidence of the successful application of co‐design methods within head and neck cancer (HNC) service redesign and research [16, 17], demonstrating the value of partnership working. Experience‐Based Co‐Design (EBCD) methodology, in particular, has been used successfully, with studies demonstrating how EBCD's structured focus on experiential ‘touchpoints’ and equal partnership working can generate innovative and meaningful solutions [14, 15].

This research aims to address gaps in education and training for community HCPs using EBCD principles to develop a prototype education and training package tailored for the needs of PwL.

## 2 Materials and Methods

### 2.1 Approach

**Figure 1:**
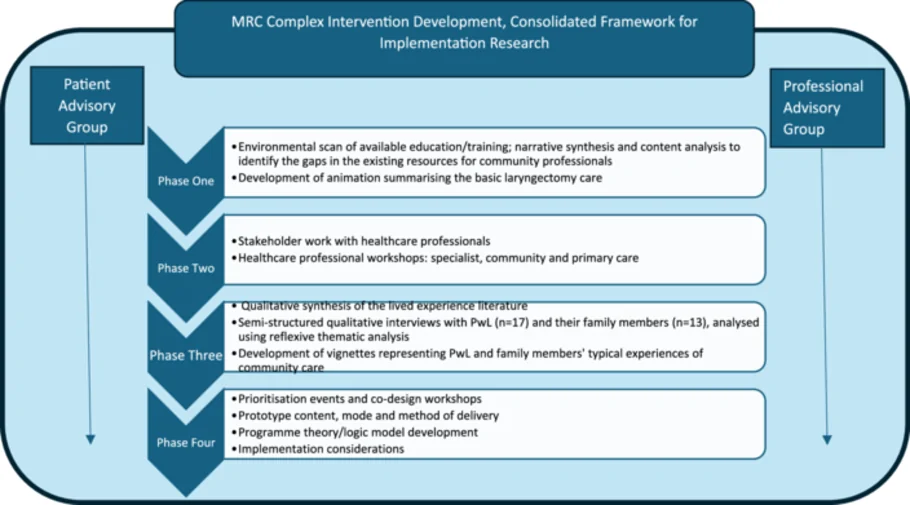
Research roadmap underpinning co‐design.

This work forms part of a larger body of research, underpinned by the Medical Research Council's (MRC) Complex Intervention Framework [18], and supported by the Consolidated Framework for Implementation Research (CFIR) [19], represented in Figure 1. The environmental scan and literature synthesis have been previously reported [13, 20].

EBCD is a participatory action design research method [21], underpinned by principles of user‑centred design, learning theory, and narrative‑based approaches to change [22]. EBCD is a flexible approach to co‐design, structured around six core steps [21]. For the project, key EBCD principles were selectively adapted to support the prototype development. The principles were modified to support a research approach that extended beyond one service or context to achieve a more nationally representative perspective, while also ensuring that all PwL were able to participate fully in the study. Modifications were made whilst ensuring alignment with the core principle of EBCD, that those with lived experience remain central to decision making [23].

Table 1 provides an overview of EBCD with adaptations made. The activities reported in the current paper are in the shaded boxes.

**Table 1:** Overview of EBCD stage and adaptations.

Experience‐based co design stage	Overview	Adaptations
One: Project set up	Observation within local service	Environmental scan and observations at three head and neck units
Two: Staff experiences	Observation and interviews with staff working within local service	Healthcare professional stakeholder work and two staff workshops recruited nationally
Three: Patient and carer experiences	Video interviews with patients/carers from one local service	Qualitative literature review and empirical research: semi‐structured qualitative interviews with patients/carers from three UK head and neck units. Audio‐recorded as per guidance from expert patient group
Four: Prioritisation events	Patient and carer event and staff event independent of one another to discuss priorities for improvement based on a ‘catalyst’ film of patient experiences	Patient, family, professional vignettes and animation used in replacement of the catalyst film
Five: Design groups	Joint with patients and staff to work on identifying a solution to the priorities for improvement	One priority focussed on to develop a scalable prototype intervention
Six: Celebration event	Reviewing final output	Virtual event in replacement of in‐person due to geographical spread of participants

This paper focusses on methods and results from stages four – six. Methods and results of the underpinning work represented in stages one – three are briefly reported for context, with relevant published findings (from stages 1 and 3 to date) referenced where appropriate.

### 2.2 Underpinning Work (Stage One – Three)

Stage one included empirical research: critical review of the available education and training resources using an environmental scanning methodology [13], as well as observations (by LJW, a Speech and Language Therapist with experience in HNC) at three UK head and neck units.

Stage two comprised extensive stakeholder work with HCPs and two virtual staff workshops with HCPs from across specialist, community and primary care sectors, recruited nationally via stakeholder networks. Workshops explored views and experiences of community laryngectomy care, and knowledge and skills needed to provide community‐based laryngectomy care. A topic guide was used. Two independent researchers supported workshops through observations, note‐taking and joint analysis, using rapid research and evaluation lab (RREAL) sheets [24]. RREAL sheets are used as a triangulation tool to enable real‐time data collection, synthesis and analysis [24]. The sheets used in these workshops were developed based on previous stages in this research, and included topics linked to views, experiences, knowledge and skills of community‐based laryngectomy care. Each researcher had their own RREAL sheet which was submitted to the lead author on completion of the workshops for analysis and to develop the final analytic themes. Stage three included two studies: a qualitative synthesis of the lived experience literature [20], and semi‐structured qualitative interviews with PwL and their families recruited from three UK units [25]. Following informed consent, interviews were conducted either in‐person or virtually by the lead author using a pre‐designed topic guide. All were audio recorded, transcribed verbatim, anonymised and iteratively analysed using reflexive thematic analysis [26].

This work formed the foundation for stages four ‐ six: prioritising events and co‐design. A ‘back to basics’ animation (developed jointly with PwL and HCPs) as an output of the environmental scan study (stage one), and vignettes of a person with a laryngectomy, family member and a district nurse were designed as an output of the work with HCPs (stage two) and qualitative interviews (stage three). Vignettes were initially designed by the lead author (LJW) and developed following feedback from co‐authors and the patient advisory group (PAG). Audio and written versions (supplementary materials) were developed to ensure they were widely accessible.

### 2.3 Prioritisation Events (Stage Four)

Two prioritisation events were conducted: one with PwL/families and one with HCPs. Participants were recruited from stakeholder networks, laryngectomy clubs (patient support groups) and previous participants from the stage three interview study who consented to further contact about the research. Eligibility criteria are detailed in Table 2.

**Table 2:** Study eligibility criteria.

Participant group	Eligibility criteria	Exclusion
PwL	– PWL stable in the community, for example, not experiencing any treatment side effects which would impact their ability to participate	– PWL unable to provide informed consent
	– Living within the United Kingdom	– PWL with cognitive impairments which would impact on their ability to provide informed consent, for example, living with advanced dementia
	– Aged 18 years or over	
	– Sufficient command of English to participate in workshops and understand participant information sheets	– PWL without sufficient command of English which would impact on their ability to fully participate
Family members	– Family member or significant other of PWL (alive or deceased) living within the United Kingdom	– Family members unable to provide informed consent
	– Aged 18 years or over	– Family members with cognitive impairments which would impact on their ability to provide informed consent, for example, living with advanced dementia
	– Sufficient command of English to participate in workshops and understand participant information sheets	– Family members without sufficient command of English which would impact on their ability to fully participate
HCPs	– HCPs in the UK from any of the following groups: HNC HCPs, for example, speech and language therapists, HNC surgeons; community HCPs, for example, GPs, DNs; clinical managers, laryngectomy clinical specialists working for laryngectomy industry partners	– HCPs without sufficient command of English which would impact on their ability to fully participate in the workshops
	– Sufficient command of English to participate in workshops and understand participant information sheets	– Unqualified HCPs with no experience of laryngectomy care

Prioritisation events were led by LJW, with facilitation from HCPs with HNC research experience. The event for PwL and family members was in person, as per their preference, at a local community centre. Communication needs and preferences were discussed with PwL before the event to ensure to ensure they felt comfortable, supported, and able to participate fully. The HCP event was virtual due to geographical spread and clinical commitments. The events began with an overview of EBCD principles, clarification of participant roles and expectations, agreement of ground rules, and activities to support engagement and a shared understanding. Events aimed to identify and agree priority areas for change in relation to laryngectomy support in the community.

Both prioritisation events followed the same format and were divided into two sections. Section one focussed on sharing underpinning work (stage one – three) to identify experiential touchpoints [21], gaps present in current laryngectomy care and education and training for HCPs. The animation and written/audio recorded vignettes of PwL, family member and district nurse were used to facilitate discussions about their care experiences and research findings of stages 1 – 3 for workshop attendees.

Section two focussed on generating a list of priority areas for action. Reflecting on the work done in section one, participants were asked to identify priority areas for change [21]. Priority areas were recorded in writing. All participants prioritised areas in order of importance using dot voting [27]. Grounding priority‐setting in the research findings ensured that agreed priorities were evidence‐informed [23].

Events were audio‐recorded, and facilitators took field notes. Principles of thematic analysis were applied after each event to generate a consolidated set of themes that captured the priority areas. Themes were reviewed by key stakeholders from the PAG, healthcare professional advisory group (HPAG) and research team (co‐authors).

### 2.4 Co‐Design (Stage Five)

Three co‐design workshops were conducted to address the top priority area for change and iteratively and collaboratively design the prototype content. Workshops were iterative, consistent with established principles of complex intervention design [28]. Workshop design was discussed with the PAG, HPAG, research team and a co‐design expert.

Participants from the interview study and stage four events, as well as stakeholders from the HPAG, were invited. Snowball sampling was used to recruit additional participants to ensure an equal number of PwL to professionals and enhance diversity. Participants were asked to commit to one workshop. Recruitment fliers were re‐distributed locally and nationally via laryngectomy groups including the Swallows [29], Life after Lary [30] and two laryngectomy industry companies [31, 32]. A designer with expertise in digitising healthcare interventions was invited to attend via snowball sampling from LJW's academic network. The designer attended all workshops in an observational and consultative capacity, with an eye to future operationalisation of the prototype.

Workshops were conducted in person, in line with guidance from the PAG and HPAG, and were held in a neutral, non‐clinical setting. Travel costs were fully reimbursed, and a voucher was provided to all participants, including HCPs, as an honorarium for their time. As per the prioritisation workshops, communication needs and preferences were discussed with PwL before each workshop, with feedback sought to ensure participants felt adequately supported and comfortable to participate in future workshops.

Each workshop lasted approximately two and a half hours and had their own distinct aim and activities (Table 3). Workshop plans and summaries were shared via email with participants.

**Table 3:** Co‐design workshops: focus, aim, activities and analysis/frameworks.

Workshop	Focus	Aim	Key activities	Analysis/frameworks
Workshop One	Building the Foundation	Develop programme theory and design content	Research summary and reflection; logic model development; topic and delivery planning	Content analysis
Workshop Two	Refining the Design	Review design content and determine delivery	Review prototype; explore digital learning styles; integrate clinical scenarios	Thematic analysis
Workshop Three	Testing and Implementation	Finalise training/education prototype and address barriers/facilitators	Review prototype; test digital version; consider implementation factors	Thematic analysis Consolidated Framework for Implementation research

Workshop one focussed on building the foundations for the prototype, including developing the content, programme theory and logic model [33] through use of a context mapping tool [34]. The context mapping tool supported participants to generate context‐mechanism‐outcome ideas within their groups. A worksheet was used to support this, with facilitators guiding the activity with starter questions, for example: ‘We are going to create clear statements about how things might work. Each statement will explain the situation (context), what happens in that situation (mechanism), and what the result could be (outcome).’ Ideas from each group were reviewed individually before grouping together. These were informally checked with participants to ensure the summary reflected their ideas. Participants reviewed the summaries to support development of the programme theory. Workshop two focussed on refining the content and design of the prototype, including the method of delivery, digital usage behaviours and learning considerations. Vignettes and clinical scenarios (supplementary materials) supported this by enabling participants to test ideas against realistic situations and identify design features needed to ensure usability and relevance in real‐world care settings.

Workshop three moved into ‘testing’ (seeking reactions to, and feedback on) an interactive digital wireframe of part of the prototype (using conversational artificial intelligence) and implementation considerations based on the CFIR [19]. Specifically, the CFIR was used to develop questions to explore the barriers and facilitators to implementation, for example, ‘What might the barriers be to implementing the prototype? Areas to think about include people, environment, cost, resources’. Questions to help guide planning, adaptation and equitable implementation were also developed using the CFIR, for example, ‘What impact might the prototype have? Areas to think about include the economy, systems, social, environment.’. Any suggestions that fell outside the scope of the project, such as proposing resources intended for use beyond the target population of community HCPs, are not reported within this paper.

Participants were split in advance into three mixed groups for activities to ensure that PwLs’ voices and experiences were represented in each group. Workshops were facilitated by three clinical‐academics (LJW, and two other clinicians with expertise in HNC research). Workshops were audio‐recorded to support field notes, data collection and analysis.

A 4‐week interval between workshops allowed preliminary data analysis from earlier workshops to inform subsequent workshops. Analysis (inclusive of audio‐recordings, field notes and materials used to support workshop activities) – which was reviewed with the research team to enhance rigour ‐ drew on a combination of approaches including content [5, 35] and principles of thematic analysis [26] as appropriate based on the nature of the data generated and the workshop aim (Table 3). The choice of analytical approach was guided by the purpose and nature of the data. Content analysis was applied to data concerning the intervention content, required materials and resources as this facilitated categorisation of information needed to inform prototype development. Thematic analysis was used to explore the barriers and facilitators to implementation, providing a deeper understanding of factors influencing intervention delivery. GUIDED [36] and TIDieR checklists [37] were used to ensure rigorous and transparent reporting. The final draft of the prototype was reviewed by the research team, PAG, HPAG and research participants prior to finalisation.

### 2.5 Stage Six

The celebration event was held remotely by video call. Those who were unable could provide written correspondence (email). Feedback and comments from both methods were gathered and incorporated prior to finalising the prototype.

### 2.6 Ethical Approval

Ethical approval for the study was provided by Yorkshire & The Humber—Leeds West Research Ethics Service Committee in June 2024 (Reference 24/YH/0074).

## 3 Results

### 3.1 Participants

Twenty‐eight participants took part in the events and/or workshops (see Table 4 for events and workshop attendance, Table 5 for participant baseline demographics). There was overlap in attendance at the events and workshops, with eight participants attending events only, eight participants attending workshops only and twelve participants attending both. Pseudonyms were used for all participants. To note, the GRIPP 2 short form [38] has been used to report patient and public involvement (see appendices).

**Table 4:** Participant attendance at prioritisation events and co‐design workshops.

Event/workshop	PwL (n = 8)	Family member (n = 5)	HCP workplace setting (n = 15)	HCP role (n = 15)	Geographical location
Event: PwL and families	5	3			8
	Male, 4	Male, 1			Northeast, 8
	Female,1	Female, 2			
Event: HCPs			12	12	12
			Acute, 6	Specialist nurses, 2	Northeast, 7
					Northwest, 1
			Primary, 1	Community nurses, 6	East England, 2
			Community, 4	Speech and language therapist, 3	Yorkshire, 1
			Mixed, 1	GP, 1	Republic of Ireland, 1
Co‐design workshop 1	3	3	9	9	15
	Male, 3	Female, 3	Acute, 4	Specialist nurses, 2	Northeast, 11
			Primary, 1	Community	Northwest, 1
			Community, 2	nurses, 2	Yorkshire, 1 Yorkshire and Northeast, 1 East England, 1
				GP, 1	
			Mixed, 2	Speech and language therapist, 2	
				Cancer care co‐ordinator, 1	
				Industry, 1	
Co‐design workshop 2	5	3	5	5	13
	Male, 5	Female, 3	Acute,3	Specialist nurses, 1 Community nurses, 1	Northeast, 11
			Primary, 1	GP, 1	Northwest, 2
				Speech and language therapist, 1	
			Community, 1	Cancer care co‐ordinator, 1	
Co‐design workshop 3	6	3	7	7	16
	Male, 6	Female, 3	Acute, 3	Specialist nurses, 1 Community nurses, 3 Speech and language therapist, 1	Northeast, 12 Northwest, 2 Yorkshire and Northeast, 1
			Community, 3	Cancer care co‐ordinator, 1	Scotland, 1
			Mixed, 1	Industry partner, 1	

**Table 5:** Study participants baseline demographics.

Demographics	PwL (n = 8)	Family member (n = 5)	HCP (n = 15)	
Age (years)	40‐49 (1)	40‐49 (1)		
	50‐59 (1)	50 – 59 (0)		
	60‐69 (3)	60 – 69 (1)		
	70‐79 (2)	70‐79 (3)		
	80+ (1)			
Gender	Male (7)	Male (1)	Male (5)	
	Female (1)	Female (4)	Female (10)	
Ethnicity	White British (8)	White British (5)	Not recorded	
Region	Northeast (7)	Northeast (5)	Northeast (8)	
	Scotland (1)		Northwest (2)	
			Yorkshire (1)	
			Yorkshire and Northeast (1)	
			East England (2)	
			Republic of Ireland (1)	
Social deprivation level	1‐5 (5)	1‐5 (2)		
*Taken from English Indices of Deprivation* *	6‐10 (1)	6‐10 (1)		
	Unknown (2)	Unknown (2)		
Education	Secondary education (4)	Secondary education (3)		
	Higher level qualification e.g., diploma (2)	Degree/Masters (2)		
	Degree/Masters (2)			
Employment status	Employed (1)	Employed (1)		
	Unemployed (1)	Retired (4)		
	Retired (6)			
Time since laryngectomy (years)	< 1 (2)			
	1‐5 (4)			
	10+ (2)			
Communication method	Silent articulation (1)			
	Tracheoesophageal speech (7)			
Distance from treating centre (miles)	< 5 (2)			
	< 10 (3)			
	10+ (1)			
	Unknown (2)			
Co‐residing with person with laryngectomy		Yes (5)		
Relationship to person with laryngectomy		Spouse (5)		
Length of relationship		21+ years (5)		
Profession			Specialist nurses (2)	
			Community nurses (7)	
			Speech and language therapist (3)	
			GP (1)	
			Cancer care co‐ordinator (1)	
			Industry (1)	
Clinical setting			Acute (5)	
			Community (7)	
			Primary (1)	
			Mixed (2)	
Years of clinical and laryngectomy experience			Range: < 1‐21+	Range: < 1‐21+
			Median: < 20	Median: < 20

* *English indices of deprivation – GOV.UK.*

### 3.2 Prioritisation Events

#### 3.2.1 PwL and Their Families

PwL and their families identified key experiential touchpoints within laryngectomy care in the community. They spoke about key areas within this including having the knowledge and skills to form meaningful relationships with HCPs, importance of support from family/social network, anxiety around the hospital environment post‐discharge, challenges with lack of community support, navigating services and HCP roles. Six priority areas were generated and ranked in order of importance:

1. In‐person training for non‐specialist HCPs.
2. Online tool for education and training.
3. Intensive support from specialist outreach team.
4. Point of contact for advice.
5. List of personal equipment supplies.
6. Questionnaire with PwL and their family on discharge.

#### 3.2.2 HCPs

As regards to HCPs’ reactions to the research findings, participants were struck by how overwhelming the post‐discharge period is for PwL and their families. They could appreciate how uncertain this time must be for PwL and families and how the lack of contact and collaboration with services in primary and community care was a real issue. Some participants also felt ‘sad’ and ‘frustrated’ (Sara, speech and language therapist) about how PwL and their families experienced care. Ultimately, the group wanted to identify areas to improve care and reduce the risk of HCPs ‘getting this wrong’ (George, nurse). The group generated five priorities for co‐design, ranked in order of importance:

1. Patient involvement in community‐based education and training.
2. Developing a service for joint home visits.
3. Personalised support visits.
4. National education and training for non‐specialist HCPs.
5. Laryngectomy passport.

#### 3.2.3 Integrated Themes

Integrating the findings from both events generated three overarching themes, within which the specific priorities were situated: *‘education and training for community and primary care staff’ (*the highest‑ranked priorities for both groups), ‘*outreach specialist care teams*, ‘*personalised information and transfer of care’*.

### 3.3 Co‐Design Workshops

#### 3.3.1 Agreement on Co‐Design Priority Area

Participants agreed that education and training should be the priority area focussed on in the workshops. Reasons for this included the need for community HCPs to ‘*understand the importance and basic anatomy after laryngectomy*’ (George, nurse) so that ‘*when there is an issue, there is easy information for staff to access*.’ (Christie, nurse). Participants agreed that priorities included in the other themes ‐ ‘*outreach specialist care teams*’ and ‘*personalised information and transfer of care’* should be areas for future research.

Although PwL and their families initially prioritised in‐person training for HCPs over an online tool, participants in the co‐design phase recognised that delivering an in‐person education and training model would require substantial service reconfiguration ‐ an undertaking that poses significant challenges in the current healthcare climate and risks undermining both uptake and implementation of the education and training. Participants agreed that a digital option would provide a solution to this, particularly within the work environment of the target users (primary and community care staff). They suggested that incorporation of interactive elements would support access, enhance engagement, reinforce learning, and support the practical application of skills in real‐world settings. It was jointly agreed that the intervention design should be centred on a digital solution, grounded in patient‐involvement, to support future use and implementation.

#### 3.3.2 Programme Theory

Participants collectively produced seven context ideas, thirteen mechanisms and nine outcomes (Table 6). Collective review demonstrated some cross‐over of ideas within the context‐mechanism‐outcomes. For example, in terms of outcomes all groups suggested that HCPs would feel more confident and/or supported in the community as an outcome of education and training.

**Table 6:** Context‐mechanism‐outcome statements.

Context	Mechanisms	Outcomes
Lack of information from hospital to community teams	Specialist information/discharge pack linking hospital to community care	Staff in community settings would feel more supported
Fragmentation of the workforce providing care to PwL	Out of hours management in the community	HCPs would feel more confident, supported and competent in laryngectomy care in the community
	Specialist information/discharge pack linking hospital to community care	
Lack of education for district nurses and doctors in the community	Access to training for GP and district nurses	PwL would have more confidence in their GPs reviewing their stoma/valve rather than leaving, ignoring or deflecting
Lack of consistent guidance for PwL and community staff	Tailored information package from a trusted healthcare professional	HCPs in the community would be more confident working with PwL
	Better support for PwL and community care	PwL would feel safe at home
Poor holistic team working between hospital and community staff	Seamless discharge summary including an emergency healthcare plan, laryngectomy passport, guidance sheet with troubleshooting advice and information about where to go for help ‐ standardised information for laryngectomy	PwL would have appropriate information at the right time
	Better communication between hospital and community care	PwL would have a point of contact
Lack of knowledge, skills and confidence of community staff when working with PwL	Streamlined education package, including annual reviews and online updates	HCPs in the community would be more confident working with PwL
	Different formats of training (do/don't document on discharge, virtual reality for visualisation of training)	HCPs in the community would be more confident working with PwL
	PwL delivered in‐person training	PwL would have more confidence in HCPs in the community
	Points of contact for community staff and PwL to access support/information	PwL would have more confidence in HCPs in the community and HCPs in the community would be more confident working with PwL
Lack of confidence from PwL towards community HCPs	Resources for education (adaptable for the user i.e., learning type/technology skills and access; broken down into sections so can access what you want when you want it)	PwL and their families could access resources independently at home

Overall, the group agreed that the following context‐mechanism‐outcome statement fit the aims of the co‐design workshops and overall research aim, *‘There is a lack of knowledge, skills and confidence of community staff when working with PwL. If there was a streamlined education package, including annual reviews and online updates, then HCPs in the community would be more confident working with PwL.’*

**Figure 2:**
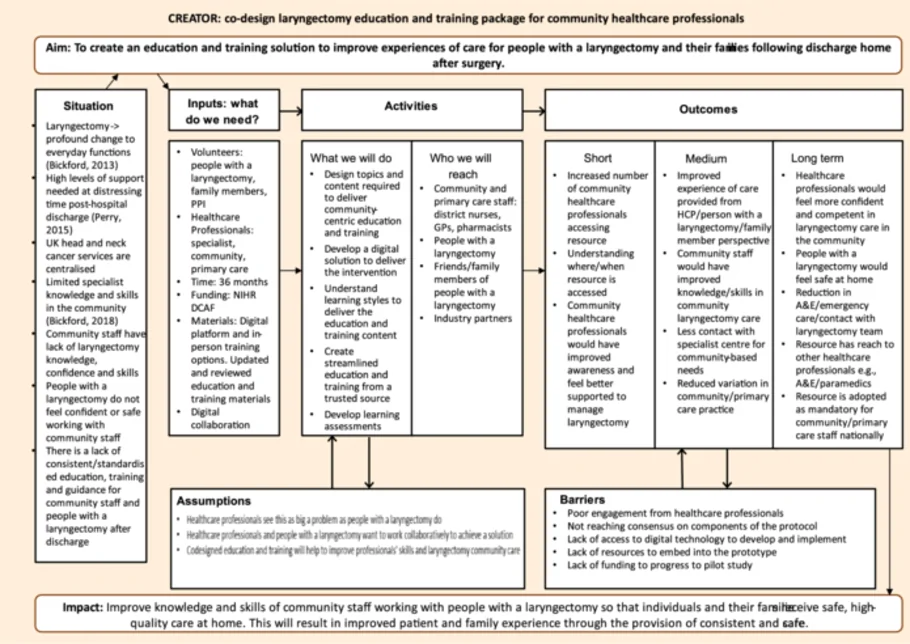
Preliminary logic model.

Suggestions for how to implement the programme theory were reviewed collectively, resulting in seven resource or staff ideas, nine risks and/or challenges with and without the prototype, and nine points for measuring success. These have supported the iterative development of the logic model (Figure 2).

In relation to resource/staff ideas, participants suggested investment in education and training, improving staff attitudes and awareness across inpatient, outpatient, and community settings, provision of appropriate equipment for community staff, the introduction of a dedicated laryngectomy care coordinator, increased allocation of time and funding, a single point of access for support and information, and regularly updated resources.

Participants highlighted several potential risks/challenges, including increased hospital admissions, unsupported care, poor care resulting from a lack of laryngectomy‐specific support, patient safety risks, inconsistencies in staff knowledge and practice, information overload, lack of personalised care, impact on clinical care, and recognised national variation in laryngectomy pathways.

Suggested measures of success included successful prototype implementation, improvements in community and patient outcome measures, reductions in accident and emergency attendances and emergency care use, increased satisfaction with healthcare services, monitoring the number of individuals accessing the prototype, identifying where and by whom the training is being used, gathering experiences from PwL, families, and HCPs, adoption of basic laryngectomy training as a mandatory requirement, and extending the reach of the prototype to other professional groups, such as paramedics.

Drawing on this cumulative work, the preliminary programme theory articulates how an education and training intervention for healthcare professionals supporting people with a laryngectomy and their families in community may contribute to meaningful change, and the conditions under which such change is most likely to occur. The theory suggests that an evidence‐informed, co‐designed, and context‐sensitive educational approach can enhance healthcare professionals’ knowledge, confidence, and understanding of laryngectomy care. In turn, this may support improved decision‐making, communication, and care delivery, with the potential to improve patient and family experiences and outcomes, while reducing avoidable reliance on specialist services.

#### 3.3.3 Digital Usage and Design Principles for Effective Learning

Participants discussed how they would envisage delivery of a digital package, including structure and personalisation within the package.

An introduction video with signposting to the digital content as an entry into the package was suggested. This could be followed by a series of questions for the user to answer to take them to the relevant sections for their learning and context.

Participants designed the materials, accessibility and topic‐specific design. Within this, participants discussed what would be required to deliver the agreed content, considering necessary resources to support effective implementation. Participants suggested video and images would be beneficial as an inroad to the content. The potential for virtual reality in the delivery of some content, such as valve and stoma management, was also discussed as an option. Voice‐overs were suggested; with the recommendation that psycho‐social and communication content be delivered by PwL.

A wide variety of resources to support learning, drawing on both digital tools (such as chat functions, webinars, online events, virtual reality, and internet searches), coupled with more ‘traditional’ formats (including books, written text, workbooks, visual materials, and lived‐experience stories) were suggested. Options to digitise more ‘traditional’ formats were explored, including the value of digitised written information, but participants suggested this needs to be succinct and digestible within the realities of clinical work. Terminology should be clear and consistent to support implementation of learning. Practical learning opportunities and strategies such as quizzes or mid‐way questions were also valued for reinforcing understanding.

Three factors were identified as central to effective learning: ease of access, visual appeal and resource credibility. Several barriers to widespread uptake were highlighted, ranging from everyday challenges such as limited time, lack of support, and content that felt unrelatable, to digital‐specific obstacles including poorly designed/overly complex websites, paywalls and large blocks of text that were visually difficult to process. Together, these insights illustrated the importance of promoting inclusivity by presenting accessible, engaging, and trustworthy learning materials that accommodate diverse preferences while minimising practical and digital barriers.

#### 3.3.4 Education and Training Prototype

Participants iteratively developed the prototype education and training based on the collective research findings and insights generated during the workshop activities.

Participants suggested six main sections within the prototype: set‐up, options for use, core topics, case scenarios, conversational artificial intelligence and main menu. Two main options for accessing the educational content in the platform following the main menu and user set up were recommended: direct through the topics presented in tiles, or via a chatbot using artificial intelligence (AI) based on the research and resource content.

Participants agreed on ten topics for content development:

1. Anatomy.
2. Breathing.
3. Safety and confidence in the community.
4. Communication.
5. Valves.
6. Equipment.
7. Life after laryngectomy.
8. Eating and drinking.
9. Alert systems and how to find help.
10. General health.

Participants recommended the order to present topics in, starting from what was most important and form the basics in laryngectomy care, progressing to more complex topics. Participants utilised the vignettes and clinical scenarios to tailor elements of the prototype content to key clinical decision points, for example, stoma care/equipment following hospital discharge. Recommended content for each topic, reflecting the identified gaps, concerns and priorities of PwL, their families and HCPs was also developed. For example, within the anatomy section of the prototype, participants suggested content such as difference between laryngectomy and tracheostomy, physiological changes, safety impact, psychological aspects and intimacy changes. Participants recommended that topic summaries and multiple‐choice questions accompany each topic to assess the users’ knowledge and highlight areas where further learning or clarification may be needed.

Participants also suggested a section for frequently asked questions, developed in response to the key issues identified throughout the research as most relevant to community laryngectomy care. As examples these include, ‘*how do I clean a stoma?*’ or ‘*where do I get laryngectomy equipment from?’*. Interactive case scenarios, using pre‐programmed responses (based on this research) to questions posed by the user, could then be available to consolidate learning from across the topics. For example, a case scenario built on a district nurse going to visit a PwL at home following discharge after surgery.

Participants also designed the methods of content delivery, including diagrams, animation, and real‐life videos within all ten topics. Core elements of the prototype were also designed, including practical tools, for example, contact routes for specialist support, and confidence building aids, for example micro‐learning opportunities. Finally, known variability in clinical advice was highlighted, and in response to this, recommendations to seek consensus on this, were advised to be incorporated into the final content.

When the wireframes (basic outline of the digital platform showing the layout, key functions, without focussing on the final look) prototype mock‐up were tested (workshop 3), participants liked the interactive element in the wireframes and suggested two options for developing this further: further refinement of the pre‐programmed questions and answers (as tested in the wireframes) to make it more streamlined, and an option for users to type their question into the core chat box to generate a response based on the content in the core programme. Participants suggested that these refinements would support ease of use and improve interactivity within the platform.

**Figure 3:**
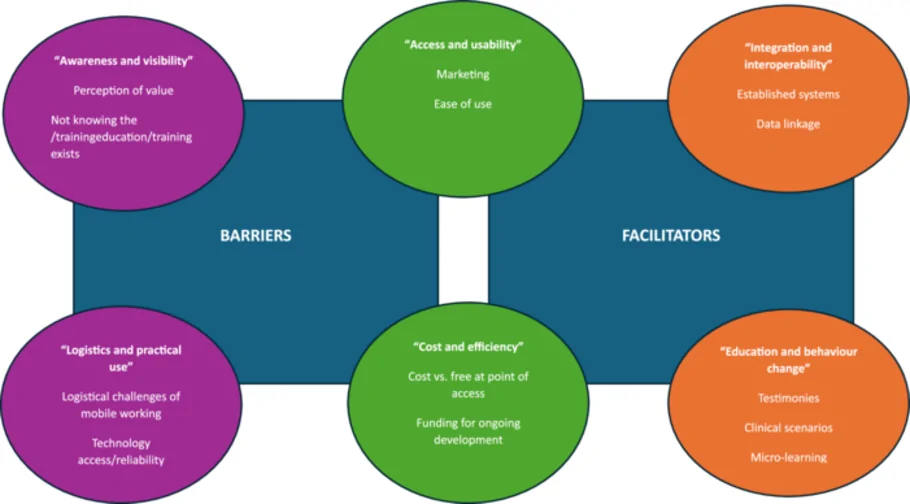
Barriers and facilitators to implementation.

The barriers and facilitators to implementation identified by workshop participants were analysed into six themes ‐ *‘logistics and practical use’, ‘cost and efficiency’, ‘awareness and visibility’, ‘education and behaviour change’, ‘integration and interoperability’, ‘access and usability’*. Themes were developed with reference to the CFIR framework to understand how the themes related to the innovation itself, the organisational and operational context (setting) and the roles and characteristics of individuals involved in implementation (Figure 3). For example, themes such as *‘cost and efficiency’* and *‘access and usability’* were primarily associated with characteristics of the innovation, reflecting perceptions of its value, practicality, and ease of use. *‘Logistics and practical use’* and *‘integration and interoperability’* related largely to the inner setting, encompassing operational processes, infrastructure, resources, and compatibility with existing systems and workflows. In contrast, *‘awareness and visibility’* and *‘education and behaviour change’, *were more closely aligned with individual characteristics and processes, highlighting the importance of knowledge, engagement, skills, attitudes, and behavioural adaptation among HCPs. Some topics within themes appear in both the barriers and facilitators, highlighting the complexity of implementing education and training within community and primary care. The dual nature of some factors can either help or hinder progress depending on how they are addressed, for example, cost.

**Figure 4:**
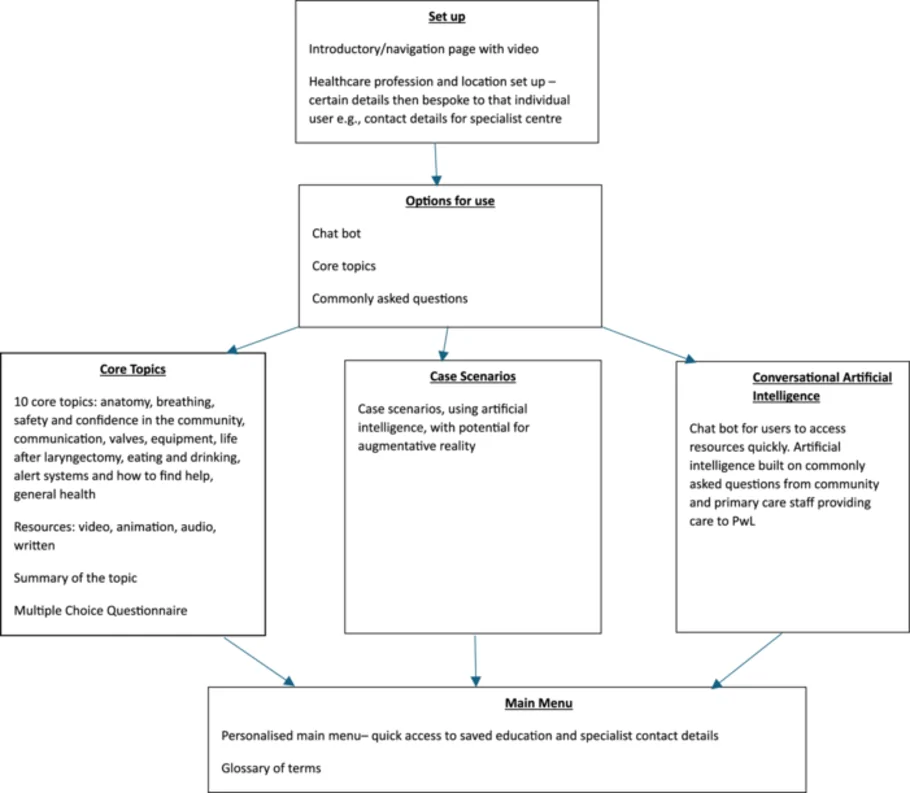
Prototype outline.

The final education and training has been drafted into a prototype for use in a digital platform. (Figure 4), along with an accompanying manual.

## 4 Discussion

To our knowledge, this is the first study to use an adapted EBCD process [21] to iteratively develop a laryngectomy‐specific prototype education and training package for use by community and primary care HCPs. This work is underpinned by guidelines and frameworks, specifically within the design phase of Complex Intervention development [18], supported by the Consolidated Framework for Implementation Research (CFIR) [19]. This approach has ensured that the prototype has been developed within a robust, evidence‑informed structure that acknowledges the realities of implementation, the needs and behaviours of end‑users and the contextual factors likely to influence adoption in real‑world settings [39]. This has also enabled an initial theory of change (see supplementary material) and a logic model [33] (Figure 2) to be established.

To ensure the methods were appropriate for both the target users (community HCPs) and recipients of care (PwL/families), adaptations were made to better align the EBCD approach to their needs and circumstances. By grounding the design in established guidelines and frameworks while incorporating lived experiences and insights, the design process has addressed the gaps in education and training for community HCPs through creating a prototype that is both practically relevant and built upon evidence. Additionally, the prototype design has been directly informed by those most affected, thereby enhancing its relevance, acceptability, and likelihood of successful adoption [23], with strong potential for testing in a future pilot study.

The decision to digitalise the prototype instead of delivering in‐person training was made collectively, reflecting the practical realities of healthcare in the UK NHS, including limitations in time, funding, resources, and workforce capacity. Evidence suggests that HCPs are accepting of digital interventions, with these offering potential to improve job performance if technology and infrastructure are supported [40]. Examples of successful implementation of digital healthcare education include the National Tracheostomy Safety Project [41] in the United Kingdom. These programmes – while not focused on laryngectomy ‐ have improved staff education, engagement and team working, reduced patient‐reported anxiety and depression, improved patient and family engagement and contributed to annual NHS cost‐savings of £275 million [42, 43].

Previous research has indicated that despite the recognised need [44], current laryngectomy education and training for community HCPs is unlikely to achieve meaningful or sustained change in practice, limiting overall effectiveness and impact [13]. This further contributes to challenges for PwL and their families in building relationships and trust with community HCPs [6, 45, 46, 47], with qualitative research among PwL emphasising that professionals require the knowledge and skills to establish safe relationships to fully meet their care needs [8, 9, 12, 20, 25]. Moves towards earlier hospital discharge in line with ERAS pathways [4, 5, 6] likely further exacerbates the situation by accelerating the transition of care from specialist services to community settings, increasing pressure on community HCPs to provide laryngectomy care at home without access to appropriate education and training. Earlier community‐based care has been successful in other clinical areas, particularly stroke [48], cardiac and pulmonary rehabilitation [49], brain injury [50] and frailty [51]. Specifically, cost savings and improved patient satisfaction have been seen in early supported discharge stroke models [48], with frailty and brain injury services providing proactive, integrative care connected to local communities [50, 51]. It is hoped that through co‐designing a prototype within a digital platform, a responsive, engaging, and accessible resource can be implemented, enabling laryngectomy services to achieve similarly good outcomes by supporting community professionals to develop the knowledge and skills needed to provide safe, person‐centred care within local communities [52].

Other areas of healthcare have described working with lived‐experience partners to co‐design education packages for healthcare professionals, with recognised benefits for the end user including promotion of more humanistic, person‐centred approaches to care [53]. Several examples are reported in literature relating to university education programmes for healthcare professionals, most prominently in nursing or social work in Europe [54] or Australia [55]. Mental health nursing programmes in Australia have successfully co‐designed postgraduate curricula with service users, demonstrating the value of genuine collaboration in the development and implementation of educational programmes [55]. More broadly, these programmes may benefit the future workforce by increasing clinical competence and confidence through enhancing core skills in communication [56], reflexivity and empathy [54].

Vignettes and clinical scenarios – which reflected authentic patient journeys, system constraints, and interprofessional experiences and interactions ‐ enabled consideration of how to tailor elements of the prototype content to key clinical decision points. Use of these materials to inform discussions helped highlight the varied expectations placed on staff and ensured reflections were anchored in genuine clinical complexity rather than hypothetical or abstract situations. This, in turn, supported identification of the most pertinent topic areas and informed the design of content for the prototype that reflects the realities of everyday practice. Achinanya et al. [17], similarly incorporated vignettes into their co‐design work with people with HNC and HCPs, highlighting how alternative stimuli can serve the same function as the EBCD ‘catalyst’ film [14] and still lead to meaningful co‐design outputs. The current work extended the mode of vignette delivery from written only [17] to audio, and incorporated clinical‐based scenarios, likely increasing the accessibility and potential impact of the stimuli.

The prototype now requires operationalisation – specifically development of the resources to use in the content delivery. It is envisaged that this will be achieved through a series of co‐development workshops. There will also be an increased focus on early evaluation through the development and testing of interactive case studies, enabling feedback to be gathered on user interaction and technology usability. The prototype intervention will then be fully integrated into the digital platform. This approach will support the refinement and development of all intervention components, in line with the Medical Research Council (MRC) guidance for Complex Intervention development [18], before scaling up to a feasibility study with evaluation within one cancer alliance. The programme theory will be tested and refined at this stage. Communication is ongoing with participants in the current study to support future prototype development, testing and evaluation, in line with the MRC Complex Interventions framework [18].

### 4.1 Limitations

The main limitation of this research was an absence of certain disciplines involved in community laryngectomy care, for example pharmacists and a limited geographical spread/gender of PwL/family members. PwL and families identified pharmacists as important points of contact, and prior studies have shown gaps in laryngectomy‐specific medication knowledge within this group [7]. Despite multiple engagement attempts, only one pharmacist contributed briefly to the HPAG, and ongoing participation was not maintained ‐ possibly due to their limited interaction with PwL. Further efforts to involve pharmacists are planned.

Most PwL and family members were from the North‐East of England and all PwL in the co‐design workshops were male. To counter this, after the work described here, the prototype was reviewed by PwL in national support groups across the Midlands and Scotland. Feedback from these groups was generally positive, with suggestions made regarding order of the content and design, for example, colour coding content.

Some participants focused on concerns specific to their stage in the laryngectomy pathway, likely linked to trauma processing [57], which occasionally influenced the direction of activities and suggestions for content. For example, suggestions were made around support that would be useful for PwL and their families; these were captured and could form the focus for future work.

### 4.2 Clinical/Research Implications

This study has demonstrated the value of equal partnership working to co‐design a prototype intervention. This process could be replicated within other areas of healthcare, for example, people living with a tracheostomy, or other HNC populations, for example, those with a feeding tube, to explore opportunities for improved integration between specialist and community services.

From a clinical perspective, clinicians may use the priorities identified in the prioritisation phase to review their current service provision and identify local opportunities for improvement. Clinicians are encouraged to do this with their service users to ensure that local population needs are met.

### 4.3 Future Research

Future research could focus on developing the prototype fully into the digital platform: for example, repeat wireframes testing of artificial intelligence components. Once this has been achieved, further funding will be secured for a pilot study using the prototype with primary and community HCPs.

Other suggestions borne out of co‐design workshops, such as stratifying content based on where PwL whom the HCP is supporting is within the laryngectomy pathway (e.g. early discharge, longer‐term survivorship, age‐related laryngectomy changes), will need to be explored. While this is novel and potentially valuable, it would require more advanced technological capability to automate and personalise content in this way. This has the potential to introduce unintended complexity, offering users multiple pathways or options that may become overwhelming, particularly for time‑pressured community and primary care professionals [58]. For these reasons, stratification represents an important but longer‑term consideration within the broader trajectory of prototype development.

#### 4.3.1 Reflections/Critical Perspective

On reflection, the workshops overall demonstrated that carefully structured activities, use of first names, non‐hierarchical interactions and open dialogue fostered an atmosphere of equal partnership and mutual respect. The importance of relationship‐building, communication support and balanced group composition in enabling meaningful participation, particularly for people living with a laryngectomy, is essential. Iterative learning between workshops led to adaptations such as mixing groups, allowing greater flexibility for informal discussion, incorporating collective feedback sessions and refining facilitation strategies to maintain focus on the project aims. While challenges remained, including managing divergent discussions, time constraints and technological issues, these experiences reinforced the value of reflexive practice in co‐design research. Overall, the process reinforces the value in participatory approaches and emphasises the importance of maintaining authentic stakeholder relationships to ensure continued engagement and avoid tokenistic involvement.

## 5 Conclusion

By integrating the lived experiences of PwL and their families with the professional perspectives of those delivering care, a digital prototype informed by identified educational and training needs within community healthcare services has been designed. The education and training prototype has potential to upskill community HCPs in laryngectomy care, with the long‐term aim of improving the experiences of care and services for PwL and their families.

## Author Contributions

**Laura‐Jayne Watson:** conceptualisation, writing – original draft, formal analysis, investigation, funding acquisition. **Linda Sharp:** writing – review and editing; supervision. **David W Hamilton:** writing – review and editing, supervision. **Vicky Thornton:** writing – review and editing, supervision. **Joanne M Patterson:** writing – review and editing, supervision.

## Ethics Statement

Ethical approval for the study was provided by Yorkshire & The Humber ‐ Leeds West Research Ethics Service Committee in June 2024 (Reference24/YH/0074). All participants provided informed written consent to participate in the study.

## Conflicts of Interest

The authors declare no conflicts of interest.

## Supporting information


Supporting File

## Data Availability

The data that support the findings of this study are available on request from the corresponding author. The data are not publicly available due to privacy or ethical restrictions.
