# Supplementary material for: Co‐Designing a Prototype Education and Training Intervention for Healthcare Professionals Working in Community Laryngectomy Care: An Experience‐Based Co‐Design Study
Source: Health Expect. 2026 Sep 14;29(5):e70870. doi: 10.1111/hex.70870 (PMC13573229; doi:10.1111/hex.70870)
Supplement: Supplementary file 1 [file HEX-29-e70870-s001.docx]

Supplementary Material

Theory of Change Diagram

The Problem

When people with a laryngectomy leave hospital, they depend on community and primary care teams who often feel underprepared and under supported. This leads to reduced patient confidence, safety concerns, and fragmented care. A lack of standardised education and training for community and primary care staff and inconsistent information flow worsens the problem.

Key Assumptions

Community/primary care staff want to provide high‑quality laryngectomy care but lack confidence.

Consistent, trusted education empowers staff to deliver safer care.

Patient and carer reassurance increases with visible staff competence.

Digital and standardised content improves equity, accessibility, and reliability.

Mechanism of Change

Training → Competence → Confidence → Better Care → Improved Patient Safety

If we create trusted, standardised, multimodal education and deliver it accessibly…

Then community staff will gain the required knowledge, confidence, and skills…

Which then leads to safer, more consistent care delivery in the home and community…

Which then makes people with a laryngectomy feel safer, more supported, and more confident…

Which contributes ultimately to improved patient experience and long‑term health outcomes.

This chain works because consistent, high‑quality training directly changes staff behaviour and competence, and this change is visible to patients and families

Enablers for Success

Co‑production with patients, carers, and frontline staff.

Strong clinical governance and endorsement.

Training delivered through multiple channels (digital, face‑to‑face, blended).

Ongoing support and refreshers for staff.

Leadership ownership and endorsement in primary care and community settings.

Feedback loops to refine materials continuously.

Evidence of Change

Staff competency assessments.

Patient‑reported confidence and safety levels.

Reduction in safety incidents or unplanned contacts, for example, rapid access ENT/SLT.

Uptake and completion of training modules.

Qualitative feedback from community teams, patients and families.

Vignette: person with a laryngectomy

Bill is a 65-year-old man. He lives with his wife June who he has been married to for nearly forty years. Bill is retired and enjoys spending time with his grandchildren, going to his allotment and catching up with friends.

Bill had a sore throat for a few weeks in April 2023. He went to his doctors in May 2023 and was given some antibiotics to take. These did not help, and Bill went back to see his doctor again. He was given some more antibiotics to take. Bill started to notice some changes in his voice and felt that the pain in his throat was getting worse. He went back to see his doctor again and was referred to the hospital to see an Ear, Nose and Throat Consultant in August 2023. After some tests including a camera, scans and a biopsy, the Consultant told Bill he had a large cancer in his voice-box. The Consultant told Bill his best option to cure the cancer would be an operation to take the whole voice-box out, called a total laryngectomy. Bill had some time to think about this with his family and had some appointments at the hospital with the nurses and speech therapists to support his decision making. He also met up with someone who had already had a laryngectomy, a man called Peter, to see what life would look like afterwards.

Bill had his laryngectomy on the 4^th^ of September 2023. He spent two weeks in hospital recovering from his operation, learning how to speak using a speaking valve and look after his laryngectomy. June came into visit every day. She also learnt some things about how to help Bill at home, for example, how to switch the nebuliser on and put the filter in his laryngectomy tube. June also watched Bill clean his valve.

Bill went home on the 19^th^ of September 2023. He felt excited to be going home from hospital and was looking forward to getting settled in and having his family come to visit.

The first two days and nights at home were a real struggle for Bill. He found it difficult to communicate with June and felt frustrated that he could not get his message across to her clearly. Bill was also feeling overwhelmed with all the new equipment he had and the routines he needed to do, sometimes forgetting how often he needed to do things and in what order he needed to do them in. Bill was also upset that he could not be as involved in family conversations when his children and grandchildren came to visit, so instead of trying to take part, Bill would sit in the background and listen. This made him feel sad and not like himself because he was always used to being in the middle of family discussions.

A few days later Bill and June had an unexpected knock at the door. June answered the door to a nurse who said she had come to see Bill. Bill was not expecting a visit from a nurse but felt hopeful that she would be able to help him – especially with checking that he was looking after his stoma and speaking valve properly. He was also hoping that he could talk to her about getting his equipment and about how he had been feeling.

But Bill became very quickly aware that the nurse did not know anything at all about a laryngectomy – she even asked Bill what that meant and if it was the same as a tracheostomy! Bill could not believe that the nurse did not know what a laryngectomy was – he would at least expect her to know the basics about what had happened to him. Bill tried to explain what had happened and what this meant for him but was finding this hard to do using his speaking valve which made him feel even more upset and frustrated. The nurse asked Bill if he needed her to do anything for him. Bill couldn’t see the point in this, so he just said, ‘no thank you, we are managing fine’. The nurse ticked a box on her sheet, left her telephone number and told Bill to call her if he needed anything.

This visit made Bill feel worse in himself and he was worried about doing things right. He did think about speaking to his GP but then thought there was no point in that because he had not even made any effort to see what had happened to him. Bill thought he would just wait another week until he went back to the hospital to see the specialist team to go through his questions as he trusted the staff there and knew that they understood and knew him well. Bill wondered whether the team from the hospital and the teams out in the community shared information or spoke to each other. He thought that it didn’t seem very likely that they did and wished that they worked more closely together so that everyone knew what was going on with him.

Vignette: family member

This is Iris, Arthur’s wife. Iris is 60 years old and works part-time. When she is not at work, Iris helps with the grandchildren and enjoys going to craft classes with her friends twice a week.

Iris knew that there was something not right with Arthur when he was going to see his GP. She kept telling him to go back to the doctors because things weren’t getting better so was pleased when he eventually got sent to the hospital. Iris thought there might be something bad happening but was not expecting Arthur to get a cancer diagnosis. She certainly was not expecting him to have to have a total laryngectomy! Iris felt so upset at the thought of this happening to Arthur but knew she had to be strong and help support him through it. She still needed to have him with her, even if he was going to sound and look different, he would still be Arthur.

The time Arthur was in hospital was hard for Iris. She missed having him at home but visited him every day. Iris really liked the staff at the hospital and felt that they asked about her too which was kind. The staff also let her watch when they were teaching Arthur how to use equipment and look after his stoma and valve. Iris found some of this quite scary but did her best to learn as she wanted Arthur to be back at home with her.

Iris was so pleased on the day that Arthur came home. The afternoon at home together was great, Iris and Arthur had some tea and cake and watched some TV before the family came to visit. Iris could see that Arthur was emotional when their children and grandchildren came – she thought this was because it was the first time they were all together at home since the operation. But then Iris noticed that Arthur was becoming more withdrawn in the conversations with the family, which was very out of character. He stopped using his speaking valve to talk and just used gestures or facial expressions to take part in the conversation. That evening and the next day he also seemed to be getting frustrated with his laryngectomy care and was often asking Iris for reassurance or help. Iris was not fully confident herself but tried her best to reassure that Arthur was doing everything that the hospital had said. Iris nearly picked up the phone to the specialist nurse at the hospital to check that they were doing everything right, but she really did not want to bother anyone as she knew how busy the staff at the hospital were. She thought Arthur had some information somewhere but didn’t know where to find anything with all the new equipment.

A day or two later Iris answered the door to a nurse who said she was coming to see how Arthur was getting on at home. Iris felt so relieved that a nurse was coming to help Arthur and thought that this would help to make them feel so much better. But Iris quickly realised that the nurse was not that confident about working with people with a laryngectomy. This made her feel uneasy and a little frustrated. Iris thought all nurses would know the basics about laryngectomy to be able to help, especially when she was coming into their own home to see him. The nurse told Iris that her and Arthur were doing well. Iris could not understand how the nurse could say that as she really didn’t seem that confident even though she was really kind and made both Iris and Arthur feel at ease.

Iris was also worried about getting the supplies for Arthur’s valve and stoma care. Iris was sure that the hospital had said something about ringing the GP to get this but could not really remember. She thought she would just phone the GP to ask for some advice. She spoke to the receptionist who said that there was nothing on the system for Arthur and there was no letter from the hospital either. Iris was sure that the hospital had said they were sending a list of everything Arthur needed so she could not understand what was going on. Iris phoned the nurse who came to see them at home. She was very kind and said she would speak to the nurse in the practice who had more experience of laryngectomy than she did to see if they could help. Iris was really pleased that she had reached out to the nurse, especially as she was trying to help them as best as she could.

Iris was looking forward to Arthur’s hospital appointment so that she could speak to the team there about how they were coping at home. It had been 9 days since Arthur had been discharged home from hospital and no-one from the team had contacted them or been to see them. Iris really wanted to get some reassurance about the routine they were following and the process for getting Arthur’s equipment. Iris thought the team at the hospital would be able to help a little more with this – she felt more confident that they know what they were doing and that they knew her and Arthur well enough to be able to support them emotionally too. Iris wondered whether there might be some extra support that could help her, and Arthur adjust to life at home and get back to doing the things that they enjoyed. Iris thought about things that might be helpful to her like speaking to a healthcare professional, getting input from social care or charities, or having a chat with another wife of someone who had been through this. Iris didn’t know whether things like this existed and was keen to see if someone could help her with that.

Vignette: healthcare professional

This is Kate. Kate is a district nurse who has worked in the community for twelve years. She enjoys going to see people in their own homes, feeling that she can really see what help and support people need to live their lives as best as they can. Kate enjoys the variety in her job and describes herself as a ‘jack of all trades’.

Kate has been asked to go and visit a new patient this morning, a lady called Susan. Kate has fifteen minutes to have a look at Susan’s records so she can prepare for her visit. She can tell from the booking that Susan has been discharged home from hospital. Kate also has another four patients to see this morning. But these are all people she has visited before, and she knows why she is going back to see them.

Kate looks on the computer at Susan’s records for some more information about her.

She can see that Susan is 55 years-old and lives about 20 minutes’ drive away. Kate comes across a discharge summary from the hospital. This tells her that Susan was discharged home from hospital four days ago after a two-week inpatient stay. The referral asks Kate to go and visit Susan to see how he is managing with her stoma care and equipment use after having a total laryngectomy. Kate has never looked after anyone with a laryngectomy for quite some time. She feels okay with knowing the basics of what a laryngectomy means but is worried about more specific things like what equipment Susan will need to use. This makes Kate feel a bit nervous about the visit to Susan – she does not want her to think that she does not have all the knowledge that she needs to help support her at home. Kate knows that if Susan thinks this, she will have less confidence in her.

Kate checks the referral to see if there is a contact name and direct telephone number at the hospital to call for some advice before she sees Susan. There is no information about who to contact on the referral. Kate also knows that there is not enough time to ring via the hospital switchboard to try and find the right person to speak to. In fact, Kate does not know who the right person to speak to would be!

Instead, Kate spends her time doing some independent research online and calls her friend, Emma, in one of the other nursing teams who used to work on a head and neck ward. Kate finds it difficult to find the right information online so spends most of her time speaking to Emma. Emma runs through the basics with Kate and gives a summary of some of the equipment she might see in Susan’s home. She encourages Kate to ask Susan about her laryngectomy to learn from her about what it is she should be doing. Emma also tries to reassure Kate that she has seen people with a laryngectomy before, even though this was nearly ten years ago.

Kate leaves for the visit to Susan feeling that she knows enough of the basics to get by and make the first visit successful. But she is aware her knowledge is rusty, and she does not like putting the expectation on the patient to have the answers. Kate wishes that she could’ve spoken to someone at the hospital before the visit. Kate also doesn’t know who Susan lives with or anything else about her which makes her feel anxious. She knows how important family members are and she wants to be able to support them through this early time at home too.

Clinical Scenarios

Bill is a 65-year-old man. He lives with his wife June who he has been married to for nearly forty years. Bill is retired and enjoys spending time with his grandchildren, going to his allotment and catching up with friends.

Bill had his laryngectomy on the 4^th^ of September 2023. He spent two weeks in hospital recovering from his operation. In this time, he started learning how to speak using a speaking valve and look after his laryngectomy. Bill went home on the 19^th^ of September 2023. He felt excited to be going home from hospital. Bill was looking forward to getting settled in and having his family come to visit.

Five days after he was discharged a district nurse came to see Bill at home. Bill was not expecting a visit from a nurse but felt hopeful that she would be able to help him – especially with checking that he was looking after his stoma and speaking valve properly. He was also hoping that he could talk to her about getting his equipment as he was not confident with how he should be doing this. Bill had also been feeling a bit low in mood and frustrated. He was hoping that he could speak to the nurse about how he had been feeling.

Gloria is a 43-year-old lady. She lives with her teenage sons. Gloria works part-time in an administrative role. In her spare time, she enjoys seeing friends and spending time with her family.

Gloria had her laryngectomy 12 weeks ago. She has just finished post-operative chemo-radiotherapy two weeks ago and is now recovering at home. Gloria is still experiencing side effects from treatment. She has pain on swallowing, the skin around her stoma is red and broken down and she has lots of thick, dry secretions in her stoma. Gloria is also struggling to communicate – she cannot use her speaking valve or her electrolarynx, so is relying on writing or her text to speech app to help. This all feels very frustrating, and she is struggling to see how things are going to get better. She also doesn’t know who she should be asking for her laryngectomy supplies.

Gloria is grateful that she is alive but is struggling with the way she looks and how much her ability to communicate has changed. This is really affecting her mood at home with her children. She is also very reluctant to leave the house.

Gloria has received a text message from her GP surgery. They have sent her a telephone appointment. This really upsets Gloria as she cannot speak on the phone yet, and even if she did, she is not sure that she would feel confident enough to talk on the telephone. There are lots of things that her GP can help with. Gloria asks her sister to call the GP to change the telephone appointment to a face-to-face appointment. This is challenging for her sister as the GP receptionist does not understand why Gloria cannot speak to the GP over the telephone. This upsets both Gloria and her sister.

Ray is an 83-year-old male. He lives with his wife Iris. Ray had a laryngectomy 8 years ago after radiotherapy did not get rid of his cancer.

Ray is independent with his laryngectomy management. He goes to the hospital 25 miles away every few months to get his speaking valve changed. Ray doesn’t mind doing this as he feels comfortable with his team at the hospital – especially as he knows they are specialists in laryngectomy.

Ray also has COPD and often has chest infections over the winter months. He manages these with antibiotics at home. One Saturday afternoon Ray started feeling generally unwell and noticed that his breathing was more difficult. He was also producing more thick, sticky secretions from his stoma. Ray had already had some antibiotics the week before, but these didn’t seem to help. He tried his nebuliser to see whether this made a difference, but he was starting to feel worse.

Iris was worried about Ray – he looked very unwell and seemed to be struggling with his breathing. She called for an ambulance to come. The ambulance turned up 45 minutes later. They checked Ray over and said he needed some oxygen. They put a face mask over his nose and mouth. Ray was unable to tell the ambulance crew what they needed to do to help him. He was trying to show them his medical information and was asking Iris to try to help to explain too. Iris was really upset so was finding this difficult. She was looking in the folder with the hospital letters in for information about Ray’s laryngectomy and what this means.

GRIPP 2 short form

| Section and topic | Item | Reported on page No |
| --- | --- | --- |
| 1: Aim | Report the aim of PPI in the study | 1  *“This research aims to address gaps in education and training for community HCPs using EBCD principles to develop a prototype education and training package tailored for the needs of PwL.*” |
| 2: Methods | Provide a clear description of the methods used for PPI in the study | 2 – 5  Methods include:   1. description of patient advisory group 2. adaptations made to ensure participation of PWL in the research, for example, m*odifications were made whilst ensuring alignment with the core principle of EBCD, that those with lived experience remain central to decision making [23].* 3. Development of animation and vignettes 4. Participation in prioritisation event and co-design workshops, for example, *communication needs and preferences were discussed with PwL before each workshop, with feedback sought to ensure participants felt adequately supported and comfortable to participate in prior to the next workshops.* 5. Equal mix of PWL, family members and healthcare professionals in each co-design workshop group to ensure representation from lived experience was equal alongside professionals 6. Review of the prototype before finalisation 7. Feedback session with PWL in the format most suited to their communication needs |
| 3: Study results | Outcomes—Report the results of PPI in the study, including both positive and negative outcomes | 6 – 13  Priority areas generated and agreed upon. Discussion with healthcare professionals in equal power situation to agree top priority area for change.  Developing CMO statements, contribution to logic model and agreement on programme theory.  Agreement on final prototype including content, materials, delivery and environment for implementation. For example, v*oice-overs were suggested; with the recommendation that psycho-social and communication content be delivered by PwL.* |
| 4: Discussion and conclusions | Outcomes—Comment on the extent to which PPI influenced the study overall. Describe positive and negative effects | 14 – 17  Design process and influence of PPI discussed.  Use of vignettes and positive impact this had on design of the intervention.  Ongoing communication with participants to inform next stage of the research.  Limitations: demographics and PPI and trauma processing. |
| 5: Reflections/critical perspective | Comment critically on the study, reflecting on the things that went well and those that did not, so others can learn from this experience | 18  Paragraph included reflecting on things that went well/not and key learning from the experience. |
